# Multistep Crystallization and Melting Pathways in the Free‐Energy Landscape of a Au–Si Eutectic Alloy

**DOI:** 10.1002/advs.201903544

**Published:** 2020-05-14

**Authors:** Güven Kurtuldu, Jörg F. Löffler

**Affiliations:** ^1^ Laboratory of Metal Physics and Technology Department of Materials ETH Zurich 8093 Zurich Switzerland

**Keywords:** Au–Si eutectic alloys, crystallization paths, fast differential scanning calorimetry, metastable melting, thermodynamics of metastable solids

## Abstract

Crystals do eventually melt if they are heated to their characteristic melting point. However, this is practically only the case for high‐temperature stable crystals, whereas low‐temperature metastable crystals generally transform, before melting, into a more stable solid during heating. Here, it is illustrated that low‐temperature crystals can, however, be melted via fast differential scanning calorimetry (FDSC), even in metallic systems where nucleation and growth kinetics are rapid. For a Au–Si eutectic alloy, various metastable and stable solid states, i.e., (Au–α), (Au–β), γ, and (Au–Si), which form under well‐controlled conditions and melt at high heating rates by preventing the metastable‐to‐stable solid phase transition, are isolated. It is demonstrated that Au_81.4_Si_18.6_ can fully melt at various temperatures, i.e., 294 °C, 312 °C, 352 °C, and 363 °C, with differing melting enthalpies ranging from 6.52 to 9.83 kJ mol^−1^. The melting and crystallization paths of the metastable solids are determined by constructing an energy−temperature diagram. This approach advances the general understanding of nucleation in metallic and other systems, and is expected to contribute to the detailed understanding of thermophysical phenomena that occur at spatially reduced dimensions and/or short time scales, for example in thin‐film deposition, nanomaterials production, or additive manufacturing.

## Graphical abstract

Metastable solid states are isolated using novel experimental strategies via fast differential scanning calorimetry and melted at high heating rates by preventing the metastable‐to‐stable phase transition in a eutectic Au_81.4_Si_18.6_ alloy. An energy−temperature diagram is constructed from their distinct melting points and enthalpies to describe the alloy's multistep crystallization and melting pathways.

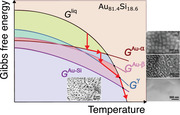

A metastable bulk liquid generally forms upon undercooling below the equilibrium melting point due to the existence of a free‐energy nucleation barrier for crystallization. The difference between the local order of the liquid and the crystal structure forms this barrier, as suggested by Frank^[^ ^1^ ^]^ to explain the large undercoolings of liquid metals observed by Turnbull.^[^ ^2^ ^]^ A metastable liquid can also form upon heating by either devitrification of a glassy phase or melting of a metastable crystal. Each crystalline solid, stable or metastable, has a specific melting point, which is the intersection of the free energies of liquid and solid. Metastable solids generally do not fully melt but transform into a more stable solid below their melting point. However, if a metastable solid can be heated at sufficiently high rates to avoid any solid−solid phase transition, its melting point and enthalpy of fusion can be measured directly. Polymorphic organic crystals, especially pharmaceutical ingredients,^[^ ^3^, ^4^ ^]^ often form at relatively slow rates by solid‐state reactions so that conventional calorimetry studies can provide insight into their thermodynamic relationships; this is essential for the development of new functional molecules. Metallic alloys also form polymorphs or metastable crystals, but their melting behavior and transition paths are difficult to identify due to their fast nucleation and growth kinetics. In this contribution, we perform fast differential scanning calorimetry (FDSC) measurements^[^ ^5^, ^6^, ^7^ ^]^ on a metallic glass‐forming system and demonstrate all possible melting and crystallization pathways for a eutectic Au_81.4_Si_18.6_ alloy. The study shows that even such a simple binary alloy can reveal multiple metastable phases that transform into each other or melt at widely different melting points. To illustrate the scope of this observation, we emphasize that the term “melting point of a crystal” is more specific than the term “melting point of an alloy.”

Binary Au–Si is a scientifically and technologically appealing system because of its deep eutectic temperature,^[^ ^8^ ^]^ its possibility of glass formation (first metallic glass discovered),^[^ ^9^ ^]^ its surface crystallization above the equilibrium melting point,^[^ ^10^ ^]^ the enhanced layering of Au–Si atoms in the liquid when in contact with the crystal,^[^ ^11^ ^]^ and the catalyzed growth of Si nanowires from Au nanoparticles.^[^ ^12^, ^13^ ^]^ Although liquid Au–Si shows a strong affinity between Au and Si atoms,^[^ ^14^ ^]^ it does not form any stable intermetallic compound, and face‐centered cubic Au and diamond‐cubic Si have only negligible mutual solubility.^[^ ^15^ ^]^ Several metastable phases were reported in the Au–Si system,^[^ ^16^, ^17^, ^18^ ^]^ but their understanding, reproducibility, and application, which are crucial for the design of new metastable materials,^[^ ^19^, ^20^ ^]^ are impeded by the complexity of their formation conditions. However, much of this complexity can be overcome by identifying the free energy−temperature landscape of crystals, which can explain the unique transformation paths between the metastable and stable phases.

**Figure** **1**a shows the melting behavior of the Au_81.4_Si_18.6_ alloy during heating at a rate of 1000 K s^−1^, revealing four distinct melting points after the formation of different solid states. The equilibrium Au–Si eutectic phase mixture melts at 363 ± 3 °C and its enthalpy of fusion is $\Delta H_{m}^{\mathrm{Au}-\mathrm{Si}}$ = 9.83 ± 0.42 kJ mol^−1^ (see also ref. [8]). It forms at relatively low cooling rates of up to 10 K s^−1^ from the melt or by a solid–solid phase transition from a metastable solid. The Au–Si eutectic microstructure, shown in Figure 1b, consists of Si particles in a Au matrix formed by a solid‐state transformation during isothermal holding of a metastable γ‐phase for 10 s at 345 °C, which is slightly below the melting point of the γ‐phase (352 °C). The Si phase in equilibrium with Au, as confirmed by scanning electron microscopy (SEM), could not be identified in the X‐ray diffraction (XRD) pattern of Au–Si in Figure 1c, probably due to the transmission geometry of the XRD analysis, the low Si composition in the alloy, and the high density difference between the Au and Si phases.

**Figure 1:**
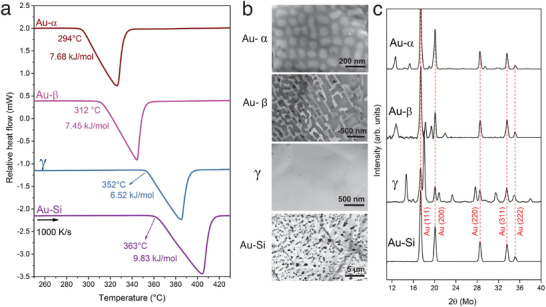
Formation of four different solid states in a Au_81.4_Si_18.6_ alloy and their melting behaviors. a) Four FDSC heating curves measured with a rate of 1000 K s^−1^, showing that Au_81.4_Si_18.6_ can have four distinct melting temperatures, which are generated by the formation of a multitude of metastable solid states (i.e., Au–α, Au–β, and γ) and the equilibrium Au–Si eutectic. It is to be emphasized that these states formed one by one in a single specimen (having a mass of 0.59 µg) after following different crystallization paths; they then melted individually at significantly different melting points. b) SEM microstructures and c) XRD patterns of the equilibrium Au–Si eutectic phase mixture, the metastable Au–α and Au–β phase mixtures, and the metastable γ‐phase.

The γ‐phase itself forms from the liquid at a cooling rate of 10 000 K s^−1^. It is a single phase with the nominal composition of the alloy. Figures 1b and 1c show the microstructure and corresponding XRD pattern of this single phase, respectively. The diffraction pattern has similarities to those of Ellner and Predel^[^ ^21^ ^]^ (18−20 at% Si, bcc, *a* = 0.5554 nm), Andersen et al.^[^ ^22^ ^]^ (25 at% Si, orthorhombic, *a* = 0.782 nm, *b* = 0.555 nm, *c* = 1.119 nm), and Krutenat et al.^[^ ^17^ ^]^ (23.3 at% Si, γ‐brass type bcc, *a* = 0.960 nm), which have almost identical, but differently indexed structures. Ellner and Predel identified the bcc phase as a substructure of Au, which may explain the Au peaks observed in the XRD pattern of the γ‐phase in Figure 1c. Heating of this metastable phase with 1000 K s^−1^ prevents its transformation into the equilibrium Au–Si eutectic and succeeds in melting the γ‐phase completely, with $T_{m}^{\gamma }\;$= 352 ± 2 °C and Δ$H_{m}^{\gamma }$ = 6.52 ± 0.28 kJ mol^−1^ (Figure 1a). These values are close to those reported by Chen and Turnbull for a single metastable phase at the Au–Si eutectic composition.^[^ ^8^ ^]^

The construction of an energy−temperature (*E*/*T*) diagram contributes to the understanding of phase transitions and relative phase stabilities in a system at constant pressure. Such *E*/*T* diagrams can be constructed using thermodynamic arguments^[^ ^23^, ^24^ ^]^ and considering that all phases are chemically identical. Eutectic phase mixtures with no primary phase have also the nominal composition of the alloy and melt at a single temperature instead of a temperature range, therefore they can also be implemented in *E*/*T* diagrams. With that the Gibbs free energy and enthalpy curves of the liquid (*G* ^liq^, *H* ^liq^), the Au–Si equilibrium phase mixture (*G* ^Au–Si^, *H* ^Au–Si^) and the γ‐phase (*G* ^γ^, *H* ^γ^) can be constructed using the melting point and enthalpy of fusion values of the solid states, as demonstrated in **Figure** **2**a. The γ‐phase has both a lower melting temperature and lower melting enthalpy than the Au–Si eutectic mixture ($T_{m}^{\gamma }<$ $T_{m}^{\mathrm{Au}-\mathrm{Si}}$ and Δ$H_{m}^{\gamma }$ < Δ$H_{m}^{\mathrm{Au}-\mathrm{Si}}$), which ensures that *G* ^γ^ does not intersect with *G* ^Au–Si^ below $T_{m}^{\mathrm{Au}-\mathrm{Si}}$. This fact verifies the metastability of the γ‐phase over the entire temperature range.

**Figure 2:**
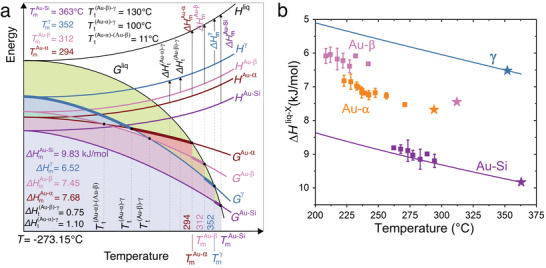
Thermodynamics of different solid states. a) Semiquantitative, schematic energy−temperature diagram of stable and metastable states in a Au_81.4_Si_18.6_ alloy. The Gibbs free energy *G* and enthalpy *H* curves were constructed using the measured melting points ($T_{m}^{\mathrm{Au}-\mathrm{Si}}$, $T_{m}^{\mathrm{Au}-\alpha }$, $T_{m}^{\;\mathrm{Au}-\beta }$, and $T_{m}^{\gamma }$) and enthalpies of fusion (Δ$H_{m}^{\mathrm{Au}-\mathrm{Si}}$, Δ$H_{m}^{\mathrm{Au}-\alpha }$, Δ$H_{m}^{\mathrm{Au}-\beta }$, and Δ$H_{m}^{\gamma }$) of the various solid states. The axes are not scaled in order to visualize the curves clearly. b) Enthalpy differences between liquid and all solid states, measured using FDSC at different temperatures during melting (stars) or solidification (squares). While the onset temperature of the endothermic peak was considered for melting, the exothermic crystallization peak temperature was considered for solidification at cooling rates between 1 and 2500 K s^−1^. Symbols and error bars represent the average enthalpy values for a temperature interval of 5 °C and their standard deviations, respectively. The continuous lines show the enthalpy differences calculated from the specific heat capacities of the liquid, stable Au–Si, and metastable γ reported by Chen and Turnbull.^[^ ^8^ ^]^ The line for γ was extended up to its reported metastable melting temperature (358 °C).

Metastable solid states other than the γ‐phase also form under different solidification conditions in Au_81.4_Si_18.6_. While a metastable Au–β mixture forms from the liquid at cooling rates between 100 and 10 000 K s^−1^, a metastable Au–α mixture forms at rates between 5 and 1000 K s^−1^. As can be seen from their microstructure shown in Figure 1b, both the Au–β and Au–α solid states are two‐phase mixtures in contrast to the single‐phase nature of γ. Rapid heating of these phase mixtures at 1000 K s^−1 [^ ^7^ ^]^ results in their melting without experiencing any solid−solid phase transition, and both Au–α and Au–β melt at different temperatures with different enthalpies of fusion. This enables us to differentiate the formation of different solid states from the FDSC heating curves, as illustrated in Figure 1a. This is verified by the SEM images and distinct XRD patterns of Au–α, Au–β, γ, and Au–Si, shown in Figures 1b and 1c, respectively. **Figure** **3** also shows the melting behaviors of various solid‐state mixtures, which can be identified via their onset temperatures of melting and their SEM microstructures.

**Figure 3:**
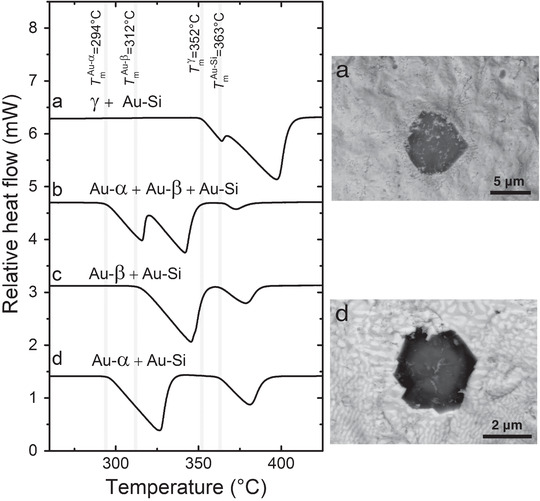
FDSC as a technique to identify specific solid states and phases from their melting behaviors during heating at a rate of 1000 K s^−1^. a) γ and equilibrium Au–Si; b) Au–α, Au–β, and Au–Si; c) Au–β and Au–Si; and d) Au–α and Au–Si. The measured melting points of the various states are indicated to compare the onsets of melting within the solid‐state mixtures. The microstructures of the solid‐state mixtures observed via SEM in backscattered electron mode are given for (a) and (d). The dark faceted particles are Si phases as determined by electron backscattered diffraction analysis.

Au–α and Au–β are eutectic phase mixtures that can be implemented in the *E*/*T* diagram together with Au–Si and γ. They have single melting points, because their FDSC melting peaks do not have any shoulder in Figure 1a and no primary phase was observed in their microstructures (Figure 1b). While fcc Au peaks are identified in the XRD patterns of Au–α and Au–β (Figure 1c), the crystal structures of α and β could not be determined from the XRD patterns. Furthermore, we would like to emphasize that a simple extrapolation of the Au liquidus line in the Au–Si equilibrium phase diagram may generate a metastable eutectic composition that differs from that of equilibrium eutectic Au–Si. However, solute trapping due to rapid solidification is likely to generate concordant compositions for the stable and metastable eutectic mixtures, as is demonstrated in Figure S1, Supporting Information.

While Au–β melts at 312 ± 2 °C with an enthalpy of 7.45 ± 0.32 kJ mol^−1^, Au–α melts at 294 ± 2 °C with an enthalpy of 7.68 ± 0.33 kJ mol^−1^. As in the case of γ, both the melting temperatures and enthalpies of Au–α and Au–β are lower than those of the equilibrium Au–Si eutectic mixture, which demonstrates their metastability for the entire temperature range (Figure 2a). However, because $T_{m}^{\gamma }\;>$ $T_{m}^{\mathrm{Au}-\beta }$ but Δ$H_{m}^{\gamma }$ < Δ$H_{m}^{\mathrm{Au}-\beta }$, a thermodynamic transition temperature $T_{t}^{(\mathrm{Au}-\beta )-\gamma }$ must exist at which the Gibbs free energy curves of γ and Au–β intersect below their melting points, as in the case of enantiotropic systems.^[^ ^23^ ^]^ As demonstrated in Figure 2a, Au–β is thermodynamically more stable than γ below $T_{t}^{(\mathrm{Au}-\beta )-\gamma }$, while γ is more stable above it. The thermodynamic transition temperature $T_{t}^{(\mathrm{Au}-\beta )-\gamma }$ is calculated as 130 °C equating the Gibbs free energy differences between the liquid and solids at $T=T_{t}^{(\mathrm{Au}-\beta )-\gamma }$. The Gibbs free energy difference between the liquid (l) and solid (s) Δ*G* ^l − s^ can be estimated using $\Delta G^{\left(l-s\right)}\left(T\right)\approx \Delta S_{m}^{s}\Delta T^{s}$, where $\Delta S_{m}^{s}$ is the entropy of fusion and Δ$T^{s}=T_{m}^{s}-T$ is the undercooling. Although this approximation assumes linear free energy curves and is generally used for small undercoolings such as $\Delta T<0.2T_{m}^{s}$,^[^ ^25^ ^]^ it can still be used here to calculate the transition temperature because of the lower specific heat difference between two solid states compared to that between a solid and liquid (a detailed illustration is given in Figure S2, Supporting Information). With $\Delta S_{m}^{s}=\Delta H_{m}^{s}/T_{m}^{s}$, the entropies of fusion for γ and Au–β are computed as 10.43 and 12.73 kJ mol^−1^ K^−1^, respectively. There also exist two other transition temperatures, which are calculated as $T_{t}^{(\mathrm{Au}-\alpha )-\gamma }=100{\;}^{\circ }C$ and $T_{t}^{\left(\mathrm{Au}-\alpha )-(\mathrm{Au}-\beta \right)}=11{\;}^{\circ }C$ (see Figure 2a). Using these values the entropy of fusion for Au–α is determined as $\Delta S_{m}^{\mathrm{Au}-\alpha }=13.54$ kJ mol^−1^ K^−1^.

The enthalpy differences between the liquid and solids can be measured not only during melting (Figure 2a and stars in Figure 2b), but also upon solidification as a function of temperature (squares in Figure 2b). The melting and solidification enthalpies for the different solid states are comparable with each other and confirm the enthalpy sequence of *H* ^Au–Si^ < *H* ^Au–α^ < *H* ^Au–β^ < *H* ^γ^. For the Au–Si eutectic mixture and the γ‐phase, the enthalpy difference is also calculated using the reported specific heat capacities of the liquid, the stable Au–Si, and metastable γ by Chen and Turnbull^[^ ^8^ ^]^ (solid lines in Figure 2b).

A time–temperature–transformation (TTT) diagram generally exhibits the formation of different solid states in the liquid as a function of isothermal holding time and temperature. To construct the TTT diagram via FDSC in **Figure** **4**a, the Au–Si alloy was melted at 450 °C, undercooled to different temperatures at a rate of 5000 K s^−1^, held isothermally for different time intervals, and then rapidly heated at a rate of 1000 K s^−1^ to identify the melting behavior of the solid states that formed during isothermal hold. Figure 4b presents a set of heating curves after the liquid was held isothermally at 247 °C for 1, 3, 5, 10 and 50 s. From these heating curves, the solid states of the various phase mixtures can be identified unambiguously by their melting point and melting enthalpy: (i) no endothermic peak due to the alloy still being fully liquid after only 1 s; (ii) only Au–α melts, indicating the sole formation of Au–α after 3 s; (iii) and (iv) Au–α and Au–Si phase mixtures melt, illustrating the transformation of Au–α into the Au–Si equilibrium phase after 5 and 10 s, respectively; and (v) sole melting of the Au–Si eutectic microstructure, demonstrating the end of the Au–α to Au–Si transformation after 50 s.

**Figure 4:**
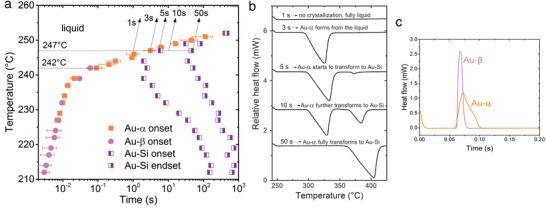
Crystallization kinetics and phase transition paths. a) Time–temperature–transformation (TTT) diagram showing the onset time of crystallization for liquid Au_81.4_Si_18.6_ isothermally held at different temperatures between 212 and 252 °C after cooling from 450 °C at 5000 K s^−1^. The time spent during cooling was not considered in this diagram. While solidification of Au–α and Au–β and their solid‐state transformation into the equilibrium Au–Si eutectic phase mixture was observed during isothermal hold, formation of the γ‐phase was not observed in this temperature interval. The phase that formed was identified by its melting point and melting enthalpy measured during heating at a rate of 1000 K s^−1^ following the isothermal hold. b) Representative data for the preparation of the TTT diagram after an isothermal hold at 247 °C. c) Exothermic crystallization peaks of Au–α and Au–β during an isothermal hold at 242 °C after melt quenching at a rate of 5000 K s^−1^.

The exothermic solidification peaks during isothermal hold also differ for Au–α and Au–β formed in the undercooled liquid. Figure 4c illustrates that Au–α and Au–β can be identified clearly during isothermal annealing by analyzing the corresponding crystallization peak characteristics. These result from different nucleation and growth kinetics, i.e., peak area (enthalpy of crystallization), peak height (nucleation and/or growth rates), and peak width (growth rate). At 242 °C, equilibrium Au–Si starts to form from Au–α or Au–β after 1–2 s of further annealing (see Figure 4a). In this time interval any presence of undercooled liquid can be excluded, because cooling rates higher than 10^6^ K s^−1^ are required to suppress crystallization in the Au–Si system.^[^ ^9^, ^26^ ^]^ Therefore, if the alloy is heated rapidly before equilibrium Au–Si forms, melting of only Au–α or Au–β occurs, as presented in Figure 1a.

As illustrated in Figure 4a, the Au–Si eutectic microstructure did not form directly from the liquid for isothermal hold temperatures below 252 °C (above which solidification was not observed at a time scale of <1000 s). Instead, Au–α or Au–β formed first in the liquid and then transformed into equilibrium Au–Si. Although the liquid was undercooled below the melting temperatures of all solid states, which makes all of them possible candidates to form from the liquid, there are specific temperature ranges at which only Au–α (243−252 °C), only Au–β (212−224 °C), or either Au–α or Au–β (224–243 °C) formed. However, this overlapping is not related to $T_{t}^{(\mathrm{Au}-\beta )-\gamma }$(=130 °C), but covers a temperature range where Au–α may transform into Au–β, but Au–β cannot transform into Au–α (see Figure 2a). An overlap of Au–α or Au–β formation, as seen in Figure 4a for isothermal measurements, is also present for cooling rates between 100 and 1000 K s^−1^ (as described above).

The solid states Au–α and Au–β have higher Gibbs free energies than γ in the temperature range between their melting points and their transition temperatures to γ (Figure 2a). **Figure** **5**a shows that Au–α and Au–β can transform into γ during heating. Here it has to be emphasized that this reverse eutectoid transformation occurs via an *endothermic* reaction. This may be surprising at first sight, but indeed is expected when considering the corresponding enthalpies as determined in this study. This is demonstrated in the energy−temperature diagram of Figure 5b, where the crystallization path for Au–α is illustrated by red arrows following the corresponding free‐energy and enthalpy curves. While the (Au–α)‐to‐γ transition enthalpy is Δ$H_{t}^{(\mathrm{Au}-\alpha )-\gamma }$= 1.10 ± 0.10 kJ mol^−1^, Δ$H_{t}^{(\mathrm{Au}-\beta )-\gamma }$ = 0.75 ± 0.15 kJ mol^−1^. These values are in good agreement with the enthalpies of fusion for the different solid states. For example, the sum of Δ$H_{t}^{(\mathrm{Au}-\alpha )-\gamma }$ and Δ$H_{m}^{\gamma }$ should be approximately equal to Δ$H_{m}^{\mathrm{Au}-\alpha }$ (1.10 + 6.52 ≈ 7.68 kJ mol^−1^), as demonstrated in Figures 2a and 5b, although all enthalpy differences were measured at different temperatures.

**Figure 5:**
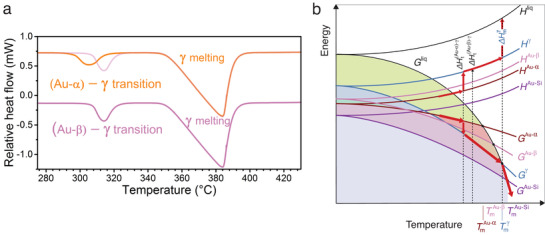
Crystallization and melting paths. a) After the formation of Au–α and Au–β, these phase mixtures could also be transformed into γ via heating at a rate of 1000 K s^−1^. This occurred via an endothermic solid‐state transformation just below their respective melting points. The heating curves also show that γ finally melts. For comparison, the heating curve illustrating the (Au–β)‐to‐γ transition is superimposed to that of the (Au–α)‐to‐γ transition. b) The (Au–α)‐to‐γ transition and subsequent γ‐melting path is demonstrated in the *E*/*T* diagram by red arrows for the corresponding free‐energy and enthalpy curves. This pathway also illustrates the endothermic nature of the (Au–α)‐to‐γ transition.

The transformation of Au–α or Au–β into γ or Au–Si demonstrates that the formation of a low free‐energy solid state from a liquid may be facilitated by the formation of a high free‐energy solid state. The *E*/*T* diagram permits to test the Ostwald step rule. According to Ostwald, the crystal phase that nucleates in the liquid is the one whose free energy is closest to the liquid, but not necessarily the thermodynamically most stable crystal.^[^ ^27^, ^28^, ^29^ ^]^ We observe that this is not the case in Au–Si. Although Au–α has a free energy closest to that of the liquid between 210 and 255 °C (Figure 2a), Au–β can also form from the melt upon isothermal hold (Figure 4a,c). This is due to the fact that the nucleation barrier also strongly depends on the solid−liquid interfacial energy, which leads for example to the fact that metastable quasicrystals with a particularly small interfacial energy can easily form from the liquid.^[^ ^7^, ^29^ ^]^ It should also be noted that the metastable solid states Au–α and Au–β are phase mixtures, whereas the metastable states in the Ostwald step rule are generally considered as single phases.

The rich variety of metastable phases present in the Au–Si system may play a key role in glass formation in the context of geometrical frustration in the liquid.^[^ ^30^, ^31^ ^]^ An undercooled liquid tends to order into a metastable or stable crystal, but the difference between the locally favored order in the liquid and the long‐range order of all possible solids is expected to retard crystallization and promote glass formation. The addition of other elements to Au–Si, such as Cu, Ag, and/or Pd, complicates the formation of any existing metastable or stable solid, so that Au–Si forms the basis for a variety of bulk metallic glasses.^[^ ^32^, ^33^, ^34^ ^]^

Metastable phases can have superior properties over their corresponding equilibrium phases and are thus potential candidates for the design of novel functional materials. However, calorimetric studies of metastable phases were so far only possible to a limited extent, due to the fact that thermodynamic parameters of metastable phases can only be determined experimentally when the measurement time scale is shorter than the lifetime of the corresponding metastable phase. We demonstrate here that this requirement is fulfilled for a Au–Si eutectic alloy using the time resolution and scan rate of FDSC. We were thus able to determine a variety of metastable solid states in this system together with their specific melting points and enthalpies. Via the construction of an *E*/*T* diagram we were also able to unravel the various metastable phases and their distinct crystallization and melting pathways. The construction of *E*/*T* diagrams is generally important to synthesize specific metastable materials and to isolate them from all other possible solid states. This work may thus set a cornerstone for detailed investigations of metastable phase diagrams, and provides novel possibilities for the discovery and design of metastable materials potentially useful in structural and functional applications.

## Experimental Section

The Au_81.4_Si_18.6_ alloy was prepared by alloying pure elements Au (99.999 wt%) and Si (99.9999 wt%) via induction melting in an argon‐purged (99.997 wt%) atmosphere. Au–Si ribbons of 30 µm thickness and 3 mm width were then produced via melt spinning. Here, the Au–Si alloy was melted in a quartz tube and ejected via high‐pressure argon onto a Cu wheel with a rim speed of 20 m s^−1^. These crystalline Au–Si ribbons had a characteristic silver color just after melt spinning, but became brittle and changed their color to yellowish brown during exposure to air at room temperature within 3 weeks. This tarnishing effect is similar to the one observed for Au‐based bulk metallic glasses.^[^ ^35^ ^]^ Thus Au–Si ribbons of another batch were immersed into liquid nitrogen immediately after production and kept there throughout the study. These ribbons did not change their color and always showed the expected melting behavior of eutectic Au–Si in FDSC (i.e., melting below 363 °C).

FDSC analysis was performed using a Mettler–Toledo Flash‐DSC 1, applying an Ar flow of 10 mL min^−1^ during the measurements. The FDSC samples were prepared by cutting small pieces from the Au–Si ribbons and then transferred onto a MultiSTAR UFS1 sensor using an electrostatic manipulator. The sample mass for FDSC,  *m* ^FDSC^ (between 300 ng and 5 µg), was determined using the melting enthalpy of the Au–Si equilibrium eutectic phase mixture, Δ$H_{m}^{\mathrm{Au}-\mathrm{Si}/\mathrm{FDSC}}$, measured by FDSC in units of Joules after the solidification occurred at 1 K s^−1^; the equilibrium enthalpy of fusion Δ$H_{m}^{\mathrm{Au}-\mathrm{Si}}$ in units of Joules per mol (9800 J mol^−1^) measured by conventional DSC; and the molar mass of the Au–Si eutectic alloy *M* ^Au–Si^ = 165.554 g mol^−1^, with the relation:

(1) $$m^{\mathrm{FDSC}}=M^{\mathrm{Au}-\mathrm{Si}}\left(\Delta H_{m}^{\mathrm{Au}-\mathrm{Si}/\mathrm{FDSC}}/\Delta H_{m}^{\mathrm{Au}-\mathrm{Si}}\right)$$

The temperature of the FDSC curves was calibrated using the melting point of the equilibrium eutectic Au–Si alloy, $T_{m}^{\mathrm{Au}-\mathrm{Si}}$= 363 ± 3 °C.^[^ ^8^ ^]^

Microstructural analysis was performed on the surfaces of the solidified alloys using a Hitachi SU‐70 scanning electron microscope, and confirmed by investigations on surfaces cleaned by focused ion beam (FIB, FEI Helios 600i system).

## Conflict of Interest

The authors declare no conflict of interest.

## Supporting information

Supporting Information
