# Supplementary material for: Multistep Crystallization and Melting Pathways in the Free‐Energy Landscape of a Au–Si Eutectic Alloy
Source: Adv Sci (Weinh). 2020 May 14;7(12):1903544. doi: 10.1002/advs.201903544 (PMC7312312; doi:10.1002/advs.201903544)
Supplement: Supplementary file 1 — Supporting Information [file ADVS-7-1903544-s001.pdf]

Supporting Information

Multistep crystallization and melting pathways in the free-energy landscape of a Au–Si eutectic alloy

Güven Kurtuldu[1]*, Jörg F. Löffler[1]

[1] Laboratory of Metal Physics and Technology, Department of Materials, ETH Zurich, 8093 Zurich, Switzerland

*Correspondence to: guven.kurtuldu@mat.ethz.ch

Supplementary Figure 1

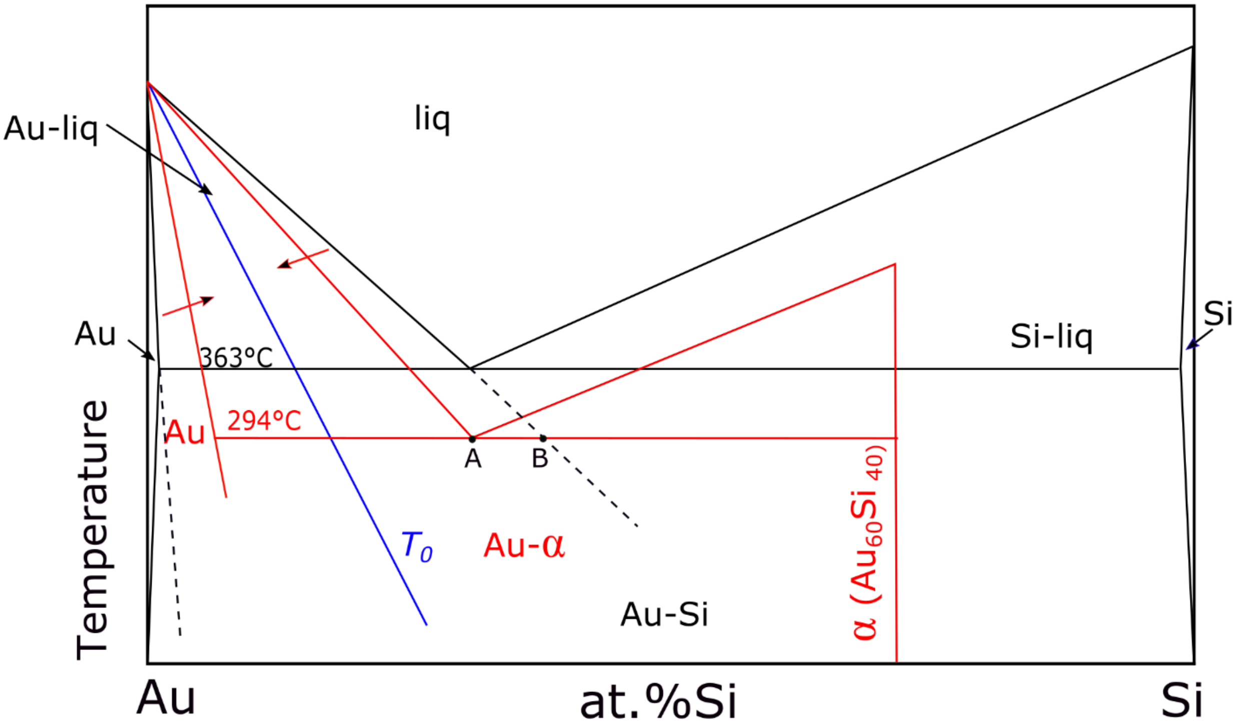

Figure S1. Schematic Au–Si phase diagram demonstrating the construction of two eutectics A and B. The eutectic B is constructed by a metastable extension of the Au-liquidus line. The eutectic A results from solute trapping, where the Au-solidus and liquidus lines approach each other (see arrows) and may converge in a T_0 line. In this case, the equilibrium Au–Si eutectic and the metastable Au–α eutectic can have equal chemical compositions.

The lack of thermodynamic equilibrium conditions in an alloy system can lead to constrained equilibrium conditions, as John Cahn stated for example in reference [1]. One example of a constrained equilibrium is a metastable phase equilibrium in the absence of a stable phase, which can be deduced from the extrapolation of equilibrium thermodynamic data. Figure S1 shows a schematic Au–Si phase diagram, where the extension of the Au-liquidus line is plotted as a

dashed line. If a metastable eutectic phase mixture exists where the Au-phase is one of the eutectic phases and metastable equilibrium conditions are satisfied, the eutectic composition will have more Si than the equilibrium Au–Si eutectic, as demonstrated by the composition B in Fig. S1.

However, in the case of rapid solidification, solute trapping may occur due to the lack of time for solute redistribution of the involved phases [1]. In the case of complete solute trapping, the free energies of the phases are equal and the solidus and liquidus lines converge to a T_0 line, which is drawn schematically for Au as a blue line in the phase diagram. (The T_0 line is not necessarily in the mid-composition of the liquidus and solidus lines.)

At intermediate solidification velocities, the liquidus and solidus lines approach each other (see arrows), as stated by Kurz and Trivedi [2], but do not form a T_0 line. When we consider high undercoolings of for example more than 100°C before the formation of the Au–α mixture in the liquid, increased solidification velocities may generate solute trapping in the Au phase, so that its Si content can be much higher than the maximum solubility of Si in Au under equilibrium conditions, which is almost zero. This leads to a shift of the Au-liquidus line and generates a eutectic between Au and α at point A. The latter is different from the eutectic obtained from extrapolation of the equilibrium Au-liquidus line (point B) and can have the same composition as the equilibrium Au–Si eutectic. In fact, composition measurements of the two phases in the Au–α mixture via energy-dispersive X-ray spectroscopy (EDS) in transmission electron microscopy (TEM) confirm that the Au-phase has 10 at.% Si dissolved and that the α-phase has the composition $Au_{60}Si_{40}$, as illustrated in the phase diagram.

Supplementary Figure 2

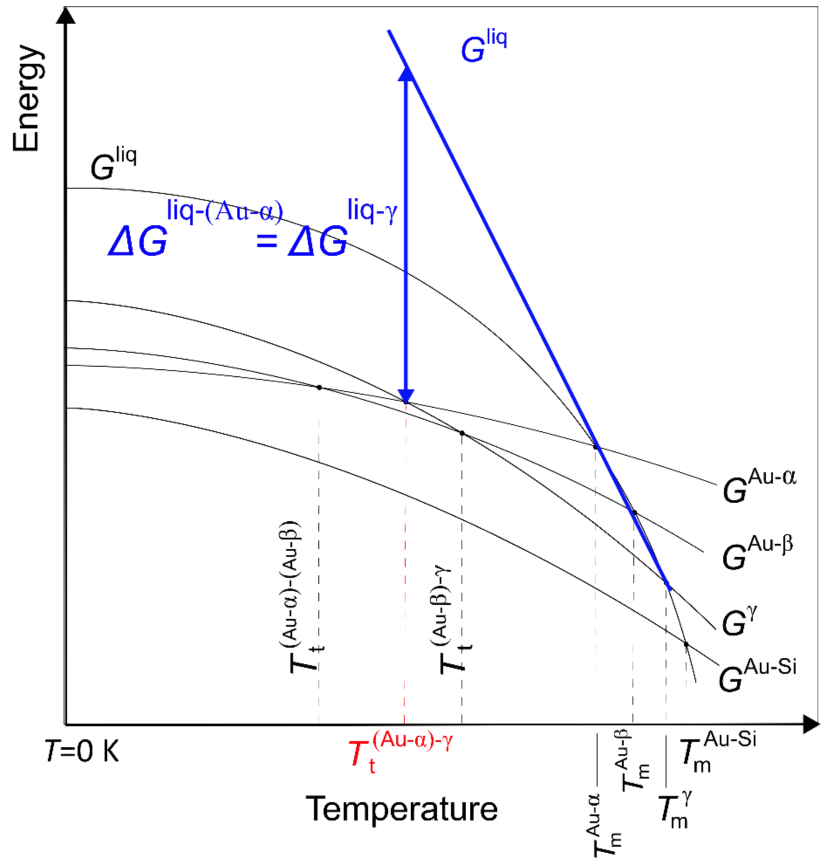

Figure S2. *E*/*T* diagram demonstrating the suitability for a linear approximation of the free-energy curves to calculate the transition temperature between Au–α and γ solids.

The Gibbs free energy difference between the liquid (l) and solid (s) $\Delta G^{\mathrm{l-s}}$ can be determined using the approximation of $\Delta G^{\mathrm{l-s}}(T) \approx \Delta S_{\mathrm{m}}^{\mathrm{s}} \Delta T^{s}$, where $\Delta S_{\mathrm{m}}^{\mathrm{s}}$ is the entropy of fusion and $\Delta T^{\mathrm{s}} = T_{\mathrm{m}}^{\mathrm{s}} - T$ is the undercooling. Although this approximation assumes linear free-energy curves, it can still be used to calculate the transition temperatures between the two solids because of their low specific heat difference. The suitability of the linear approximation to determine the transition temperatures between the solid states is demonstrated in Fig. S2. It shows that if the

slope of the liquid free-energy curve changes significantly for high undercoolings (for example at the calculated transition temperatures), the linear approximation of $\Delta G^{\mathrm{l-s}}(T) \approx \Delta S_{\mathrm{m}}^{\mathrm{s}} \Delta T^{s}$ leads to unrealistically high free energy differences between the liquid and solid states (blue line in the figure). However, the free energy differences between the liquid and different solid states are equated for the transition temperature calculations [$\Delta G^{\mathrm{l-\gamma}}(T_{\mathrm{t}}^{(\mathrm{Au-\alpha})-\gamma}) = \Delta G^{\mathrm{l-(Au-\alpha)}}(T_{\mathrm{t}}^{(\mathrm{Au-\alpha})-\gamma})$]. The temperature differences between the melting points of the solids are small, which means that the linear approximation of the liquid free-energy curve is still valid for such small temperature ranges (maximum 58°C between the γ and Au–α solid states). In addition, the slope differences of the solid free-energy curves are small due to the low specific heat differences between the solids. When we take into account these contributions, it becomes evident that the linear approximation is suitable for the transition temperature calculations.

References

[1] J. W. Cahn, *Bull. Alloy Phase Diagrams* **1980**, *1*, 27.

[2] W. Kurz, R. Trivedi, *Metall. Trans. A* **1991**, *22*, 3051.
